# Strain Tuning via Larger Cation and Anion Codoping for Efficient and Stable Antimony‐Based Solar Cells

**DOI:** 10.1002/advs.202002391

**Published:** 2020-11-23

**Authors:** Riming Nie, Kyoung Su Lee, Manman Hu, Sang Il Seok

**Affiliations:** ^1^ School of Energy and Chemical Engineering Ulsan National Institute of Science and Technology (UNIST) 50 UNIST‐gil Eonyang‐eup Ulju‐gun Ulsan 44919 Republic of Korea

**Keywords:** doping, metal chalcogenides, Sb_2_(S*_x_*Se_1−_*_x_*)_3_, Sb_2_S_3_, strain, trap states

## Abstract

Strain induced by lattice distortion is one of the key factors that affect the photovoltaic performance via increasing defect densities. The unsatisfied power conversion efficiencies (PCEs) of solar cells based on antimony chalcogenides (Sb‐Chs) are owing to their photoexcited carriers being self‐trapped by the distortion of Sb_2_S_3_ lattice. However, strain behavior in Sb‐Chs‐based solar cells has not been investigated. Here, strain tuning in Sb‐Chs is demonstrated by simultaneously replacing Sb and S with larger Bi and I ions, respectively. Bi/I codoped Sb_2_S_3_ cells are fabricated using poly[2,6‐(4,4‐bis(2‐ethylhexyl)‐4H‐cyclopenta[2,1‐b;3,4‐b']dithiophene)‐alt‐4,7‐(2,1,3‐enzothiadiazole)] as the hole‐transporting layer. Codoping reduced the bandgap and rendered a bigger tension strain (1.76 × 10^−4^) to a relatively smaller compression strain (−1.29 × 10^−4^). The 2.5 mol% BiI3 doped Sb_2_S_3_ cell presented lower trap state energy level than the Sb_2_S_3_ cell; moreover, this doping amount effectively passivated the trap states. This codoping shows a similar trend even in the low bandgap Sb_2_(S_x_Se_1‐x_)_3_ cell, resulting in 7.05% PCE under the standard illumination conditions (100 mW cm^−2^), which is one of the top efficiencies in solution processing Sb_2_(S_x_Se_1‐x_)_3_ solar cells. Furthermore, the doped cells present higher humidity, thermal and photo stability. This study provides a new strategy for stable Pb‐free solar cells.

## Graphical abstract

Strain tuning in Sb‐Chs was demonstrated by simultaneously replacing Sb and S with larger Bi and I ions, respectively. This strategy has been applied in Sb_2_(S*_x_*Se_1–_ *_x_*)_3_ solar cells, and the champion cell exhibits a PCE of 7.05% under the standard illumination conditions (100 mW cm^−2^), which is one of the top efficiencies in solution processing Sb_2_(S*_x_*Se_1–_ *_x_*)_3_ solar cells.

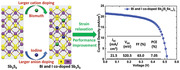

Strain is caused by distortion of the crystal structure or tensile and compressive deformation, which greatly affects the bandgap of the semiconductors, internal polarization, and the mobility of charge carriers, and thus contributes to the photovoltaic effect.^[^ ^1^ ^]^ Zhao et al. determined that the mismatched thermal expansion between the perovskite and substrate produced the strain, which caused the instability of the perovskite films and accelerated the decomposition of the perovskite.^[^ ^2^ ^]^ Wang et al. reported that the charge carrier lifetime increased with increasing tensile strain and decreased with increasing compressive strain.^[^ ^3^ ^]^ The outstanding overall film flexibility and highly stable optical response of CH_3_NH_3_PbI_3_ (MAPbI_3_) are attributed to its balanced optical response to tensile and compressive strains. The strain in the perovskite thin films can be also relaxed by alloying engineering or via continuous light illumination, which will enhance their stabilities and improve their device performance via reducing trap density and increasing carrier lifetime.^[^ ^4^ ^]^ Besides perovskite solar cells, strain is also widely studied in GaAs solar cells. Ekins‐Daukes et al. reported a strain‐balance approach to improve the efficiency of the GaAs solar cells. The tensile strain in GaAsP barriers match with the compressive strain in the InGaAs quantum wells, which overcomes the lattice‐mismatch limitation and exhibits the potential to enhance the PCE of the GaAs solar cells.^[^ ^5^ ^]^ In addition, GaP strain compensation layers have been incorporated into GaAs solar cells, which reduced strain and defects, resulting in improved device performance.^[^ ^6^ ^]^ Also, the effect of strain on device performance is investigated in organic solar cells. Lipomi et al. investigated the effect of tensile strains (≈20%) on the photovoltaic properties of organic solar cells, including poly(3‐hexylthiophene) (P3HT) and a donor–acceptor polymer whose repeat unit comprises a diketo pyrrolo‐pyrrole moiety, thiophene, thienothiophene, and thiophene (DPPT‐TT) based solar cells. They found solar cells based on both P3HT and DPPT‐TT exhibited increased open‐circuit voltages with strain.^[^ ^7^ ^]^ Although the strain is widely investigated in solar cells, it has not been studied in metal chalcogenide solar cells.

Antimony chalcogenides (Sb‐Chs), such as Sb_2_S_3_, Sb_2_Se_3_, and Sb_2_(S/Se)_3_ have attracted significant attention owing to their suitable bandgaps in the range of 1.2–1.7 eV, high absorption coefficients, excellent air/moisture‐stability, and environmentally friendly features.^[^ ^8^ ^]^ These cells are mainly fabricated by sensitized or planar architecture. Although the bandgaps and other properties of Sb‐Chs are comparable with those of metal halide perovskites, the power conversion efficiencies (PCEs) of the solar cells fabricated using Sb‐Chs are significantly lower than those of perovskite solar cells. The relatively poor PCEs have been mainly attributed to the low open‐circuit voltage (*V*_OC_) of the cells, which is because of the main energy‐loss channel induced by the strong electron–phonon interactions, trap states, and impurities.^[^ ^9^ ^]^ Recently, Yang et al. systematically studied the properties of excited‐state carriers in Sb_2_S_3_.^[^ ^10^ ^]^ Strong Stokes‐shifted photoluminescence (PL) and ultrafast picosecond carrier‐trapping processes were observed in Sb_2_S_3_; moreover, the trapped carriers were not saturated even when carrier density was as high as 10^20^ cm^−3^. These results indicated that the photoexcited carriers were self‐trapped by the distorted lattice of Sb_2_S_3_, and not by the typically assumed surface/interface/bulk extrinsic trap states. Thus, the analysis of the lattice distortion of Sb_2_S_3_ and reducing the strain is crucial for improving the performance of solar cells.

In this study, the strain tuning in Sb‐Chs was reported by simultaneously replacing Sb and S with larger Bi and I ions, respectively. Bi/I codoped Sb_2_S_3_ were prepared using an SbCl_3_‐thiourea (Sb‐TU) complex and BiI_3_ solution. As the amount of BiI_3_ added to Sb_2_S_3_ increased, the bandgap decreased slightly within the experimental range of 0–5 mol%. The performance of the Bi/I codoped Sb_2_S_3_ was highest at 2.5 mol% BiI_3_ doping and applied to Sb_2_(S*_x_*Se_1–_ *_x_*)_3_ to fabricate a device with PCE of 7.05%. The morphology and energy levels of the undoped and doped samples were investigated using field‐emission scanning electron microscopy (FE‐SEM) and ultraviolet photoelectron spectroscopy (UPS). Electrochemical impedance spectroscopy (EIS) measurements, steady‐state PL, and time‐resolved PL (TRPL) spectra were used to study the interfacial charge recombination and trap states. The interlattice distance and interface strains were investigated using high‐resolution transmission electron microscopy (HRTEM) and Williamson‐Hall (W‐H) analysis.

**Figure** **1**A depicts codoping of larger cation (Bi) and anion (I) in Sb_2_S_3_. Figure 1, 2 illustrates the XRD patterns of Sb_2_S_3_ and Bi/I codoped Sb_2_S_3_ with Bi contents in the range of 0–5 mol%. The XRD patterns of all analyzed samples were in good agreement with that of the Sb_2_S_3_ reference (JCPDS No. 42–1393). The high‐resolution XRD patterns in the 2*θ* ranges of 15°–18° and 27.5°–30° revealed that the peaks of the doped samples were shifted toward lower angles compared with those of the undoped samples (Figure 1C,D). This was ascribed to the atomic radius of Bi being larger than that of Si and indicated the efficient replacement of Sb with Bi. Figure S1 (Supporting Information) presents the direct bandgap changes in Bi/I codoped Sb_2_S_3_ with the increasing Bi content. The bandgap values were obtained in Tauc plots, which were calculated by using the UV–vis absorption spectra (**Figure** **2**C). Tauc plots are widely used to calculate the bandgaps of Sb_2_S_3_.^[^ ^11^ ^]^ The direct bandgap decreased from 1.70 to 1.62 eV as the Bi content increased from 0 to 5 mol%. The successful Bi/I codoping was also confirmed using XPS analysis (Figure 1E,F). As the doping content increased, the intensity of the peaks in the XPS profiles of the samples increased.

**Figure 1:**
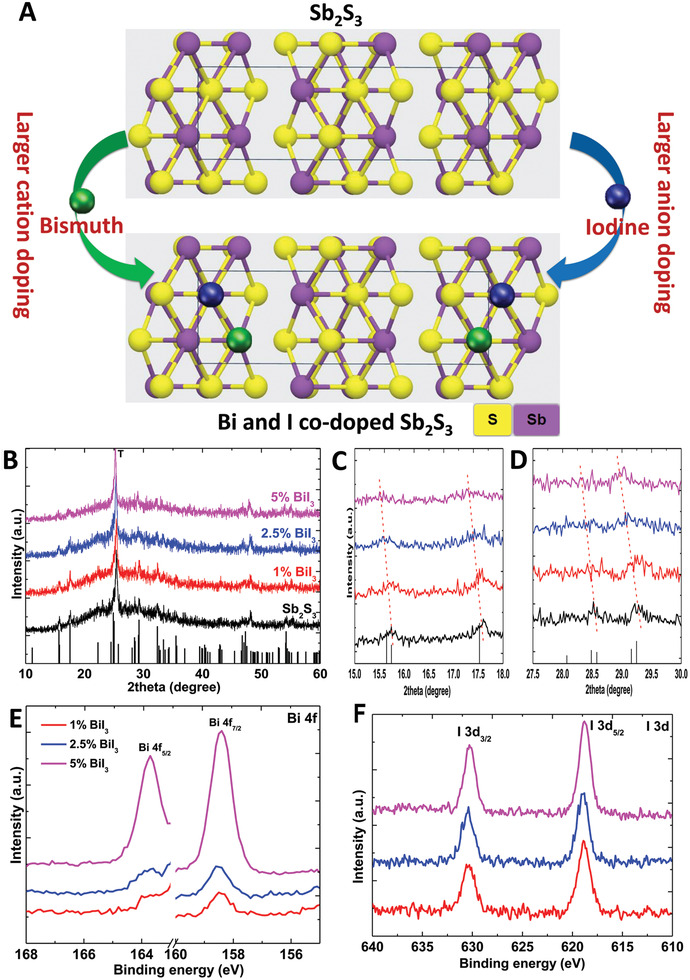
Confirmation of Bi/I codoping. A) Schematic diagram to illustrate codoping of larger cation (bismuth) and anion (iodine) in Sb_2_S_3_. X‐ray diffraction (XRD) patterns of Sb_2_S_3_ and Bi/I codoped Sb_2_S_3_ in the 2*θ* ranges of B) 10°–60°, C) 15°–18°, and D) 27.5°–30°. E) Bi 4f and F) I 3d high‐resolution X‐ray photoelectron spectroscopy profiles of Sb_2_S_3_ and Bi/I codoped Sb_2_S_3_. The standard XRD profile of Sb_2_S_3_ (JCPDS No. 42–1393) is plotted as the black bar graph in (B), and the main peak of TiO_2_ at 25.3° is marked as “T.”

**Figure 2:**
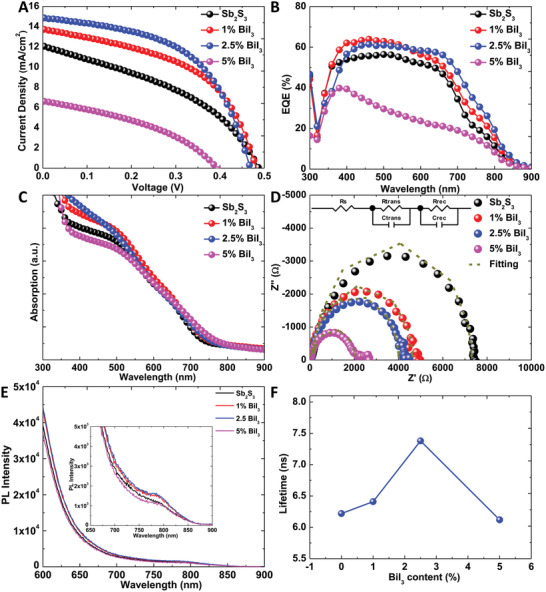
Effect of Bi/I codoping on the performance of solar cells. A) *J*–*V* curves under standard illumination conditions (100 mW cm^−2^) of AM 1.5 G and B) incident photon‐to‐current‐efficiency spectrum of Sb_2_S_3_ and Bi/I codoped Sb_2_S_3_ solar cells; Bi content in the range of 0–5 mol%. C) Ultraviolet‐visible absorption spectrum of the glass/mesoporous TiO_2_/Sb_2_S_3_ (Bi/I codoped Sb_2_S_3_) cells. D) Nyquist plots of Sb_2_S_3_ and Bi/I codoped Sb_2_S_3_ solar cells in the dark. The inset in D) is the equivalent circuit used to fit impedance curves. E) Steady‐state photoluminescence (PL) spectra and F) PL lifetime of glass/Al_2_O_3_/Sb_2_S_3_ (Bi/I codoped Sb_2_S_3_). The inset in (E) is the magnified image of PL spectra. Here EQE denotes external quantum efficiency. *R*_s_ denotes the series resistance. *R*_trans_/C_trans_ denote the resistance/capacitance associated with HTM. *R*_rec_/*C*_rec_ denote the resistance/capacitance related to the Bi/I codoped Sb_2_S_3_/HTM interface.

The surface FE‐SEM images of the glass/mesoporous TiO_2_ (mp‐TiO_2_)/Sb_2_S_3_ (Bi/I codoped Sb_2_S_3_) are presented in Figure S2A–D (Supporting Information). Their morphologies were similar with that of mp‐TiO_2_, which indicated the uniform distribution of Sb_2_S_3_ and Bi/I codoped in the mp‐TiO_2_ matrix. The corresponding energy‐dispersive electron spectroscopy (EDX) data indicated that the Sb‐to‐Bi ratios of Sb_2_S_3_ and Bi/I codoped Sb_2_S_3_ matched the stoichiometric ratios of the precursors (Figure S3, Supporting Information). Sb_2_S_3_ and Bi/I codoped Sb_2_S_3_ were used as light‐harvesting materials for solar cells in which poly [2,6‐(4,4‐bis(2‐ethylhexyl)‐4H‐cyclopenta[2,1‐b;3,4‐bʹ]dithiophene)‐*alt*‐4,7‐(2,1,3‐benzothiadiazole)] (PCPDTBT) and poly(3,4‐ethylenedioxythiophene) codoped with poly(4‐styrenesulfonate) (PEDOT:PSS) were used as the hole‐transporting material and layer (HTM(L)), respectively. The fluorine‐doped tin oxide (FTO) layer, TiO_2_‐blocking layer, mp‐TiO_2_/Bi/I codoped Sb_2_S_3_/HTM(L) layer, and Au layer can be observed in the cross‐sectional FE‐SEM image of the cell (Figure S2E, Supporting Information). The uniform morphology of the mp‐TiO_2_/Bi/I codoped Sb_2_S_3_/HTM(L) layer confirmed the efficient infiltration of the HTM(L) and uniform distribution of Bi/I codoped Sb_2_S_3_ in the mp‐TiO_2_ matrix. Subsequently, the energy levels of Bi/I codoped Sb_2_S_3_ were measured using UPS. Figure S4 (Supporting Information) illustrates the magnified images of the secondary electron cutoff and the highest occupied molecular orbital (HOMO) regions of the He I UPS spectra for the FTO/Sb_2_S_3_ (Bi/I codoped Sb_2_S_3_). The Fermi levels (*E*_F_s) of Sb_2_S_3_ and Bi/I codoped Sb_2_S_3_ with 1, 2.5, and 5 mol% BiI_3_ were 4.95, 4.79, 4.72, and 4.74 eV, respectively, and the valence band maxima (VBMs) are located at 0.97, 1.32, 1.22, and 1.27 eV below the corresponding *E*_F_, respectively. The energy levels were obtained according to these results and the material bandgaps (Figure S1, Supporting Information). As depicted in Figure S4 (Supporting Information), the conduction band maxima (CBMs) and VBMs of Sb_2_S_3_ and Bi/I codoped Sb_2_S_3_ with 1, 2.5, and 5 mol% BiI_3_ were 4.22 and 5.92 eV, 4.43 and 6.11 eV, 4.29 and 5.94 eV, and 4.39 and 6.01 eV, respectively, which matched the CBM of TiO_2_ and HOMO of PCPDTBT, respectively.

The effect of the Bi content on the performance of Bi/I codoped Sb_2_S_3_ solar cells was investigated. Figure 2A presents the current density–voltage (*J*–*V*) curves of the Sb_2_S_3_ and Bi/I codoped Sb_2_S_3_ solar cells with Bi contents in the range of 0–5 mol% under standard AM 1.5 G illumination conditions (100 mW cm^−2^), and Table S1 (Supporting Information) summarizes the corresponding performance parameters. The hysteresis in this kind of solar cell was very small (Figure S5, Supporting Information). The Sb_2_S_3_ solar cells exhibited a relatively low PCE of 2.34% with short‐circuit current density (*J*_SC_), *V*_OC_, and FF of 12.08 mA cm^−2^, 487 mV, and 39.8%, respectively. The relatively low PCE of the Sb_2_S_3_ cells was attributed to the use of commercial‐grade mp‐TiO_2_ instead of in house‐made mp‐TiO_2_ for manufacturing the cells. As the Bi content increased to 2.5 mol%, the PCE increased to 3.69%; moreover, *J*_SC_ and FF increased and *V*_OC_ slightly decreased, which could be caused by the incorporation of Bi into Sb_2_S_3_ decreasing the bandgap of the doped materials. The PCE significantly decreased when the Bi content further increased, and reached 1.01% when the Bi content was 5 mol%. To determine the reason for the change in PCE, we obtained the incident photon‐to‐current‐efficiency (IPCE) spectra of the samples, which are related to the *J*_SC_. As depicted in Figure 2B, the external quantum efficiencies (EQEs) of the samples increased and red shifted as their Bi content increased from 0 to 2.5 mol%. As the Bi content further increased to 5 mol%, the EQEs significantly decreased. The EQE spectra beyond the absorption of Sb_2_S_3_ and Bi/I codoped Sb_2_S_3_ can be attributed to the additional absorption from PCPDTBT, which have been confirmed that it can further improve the PCEs.^[^ ^8d,i^ ^]^ The EQE generally depends on the light‐harvesting efficiency and charge‐transfer yield (CTY), where the CTY is the product of the electron injection yield and charge collection efficiency. As the Bi content increased, the absorption edge increased (Figure 2C), and that was also observed in the Tauc plots of the solar cells (Figure S1, Supporting Information). The initial increase and red shift in EQE when the Bi content increased from 0 to 2.5 mol% can be attributed to the increase in the absorption edge. The subsequent decrease in EQE with the increasing BiI_3_ content to 5 mol% could be attributed to the decrease in CTY of the cells. EIS measurements were performed to investigate the CTYs of the cells. In this study, the EIS spectra were measured at an applied bias of 0.3 V, in the frequency range of 0.1 MHz to 0.1 Hz, at 10 mV AC amplitude, and in the dark. Figure 2D illustrates the Nyquist plots of the Bi/I codoped Sb_2_S_3_ solar cells. The equivalent circuit in the inset was used to fit the curves. The fitting values of series resistance (*R*_s_), the resistance/capacitance associated with HTM (*R*_trans_ and *C*_trans_), and the resistance/capacitance related to the Bi/I codoped Sb_2_S_3_/HTM interface (*R*_rec_ and *C*_rec_) are summarized in Table S2 (Supporting Information). Two obvious semicircles are observed for the Bi/I codoped Sb_2_S_3_ solar cells, which correspond to two different charge transfer or transport behaviors. The semicircle at high frequency can be ascribed to the charge transport in HTM, and the semicircle at low frequency can be attributed to the charge transfer at the Bi/I codoped Sb_2_S_3_/HTM interface.^[^ ^12^ ^]^ As the Bi content increases to 2.5 mol%, both the *R*_trans_ and the *R*_rec_ decrease, indicating more efficient charge transport in HTM and more severe charge recombination at the Bi/I codoped Sb_2_S_3_/HTM interface, which match well with the increased FF and the decreased *V*_OC_. When the Bi content further increases to 5 mol%, the *R*_trans_ increases, and the *R*_rec_ decreases, resulting in the decreased FF and *V*_OC_. These results also confirmed that the decrease in *J*_SC_ of the devices with high Bi content (5 mol%) was ascribed to their low CTY. To elucidate the cause for the differences in CTYs between solar cells, the defect states of Sb_2_S_3_ and Bi/I codoped Sb_2_S_3_ were investigated by measuring the decrease in the intensities of the steady‐state PL and TRPL of the devices. To enhance the PL intensity, the samples of glass/Al_2_O_3_/Sb_2_S_3_ (Bi/I codoped Sb_2_S_3_) are prepared. As presented in Figure 2E, a peak at about 790 nm was found for each sample, which can be attributed to Sb_2_S_3_.^[^ ^13^ ^]^ The peak shift is not obvious after incorporating BiI_3_, which might be because these peaks are relatively broad, and the doping amount is relatively low. Besides, compared to that of direct bandgap semiconductors, PL behavior in Sb_2_S_3_ (Bi/I codoped Sb_2_S_3_) is more complicated because they are indirect bandgap semiconductors. The PL intensity initially increased as the Bi content increased from 0 to 2.5 mol% and decreased as the Bi content further increased to 5 mol%. The lifetimes of the Bi/I codoped Sb_2_S_3_ samples exhibited the same trend as their steady‐state PL intensities (Figure 2F and Figure S6, Supporting Information). These results indicated that the small amount of Bi (≤2.5 mol%) that replaced Sb could reduce the defects in Bi/I codoped Sb_2_S_3_; however, the higher Bi content (5 mol%) could induce lattice distortion, which would result in the increase in defects. Therefore, the low CTYs of the devices with high Bi content (5 mol%) could be attributed to the increase in defects.

The increase in charge transfer in the cells after incorporating the optimal amount of BiI_3_ into Sb_2_S_3_ was attributed to the decrease in defects states, which might be caused by the decrease in the strains in Bi/I codoped Sb_2_S_3_. Thus, we further characterized the Sb_2_S_3_ and Bi/I codoped Sb_2_S_3_ layers using HRTEM and XRD. **Figure** 3A–sF present the HRTEM images and corresponding bright field and high‐angle annular dark‐field scanning transmission electron microscopy (BF/HAADF‐STEM) images and EDX element mappings of Sb_2_S_3_ and 2.5 mol% BiI_3_‐doped Sb_2_S_3_. The inter‐lattice spacings of Sb_2_S_3_ and 2.5 mol% BiI_3_‐doped Sb_2_S_3_ were 5.67 and 5.80 Å, respectively, which corresponded to the (020) orientation at 15.644° in the XRD patterns of these samples (Figure 1C). The inter‐lattice spacing of 2.5 mol% BiI_3_‐doped Sb_2_S_3_ was 0.13 Å larger than that of Sb_2_S_3_ owing to the replacement of Sb with Bi and the ionic radius of Bi being larger than that of Sb. The compositions of Sb_2_S_3_ and 2.5 mol% BiI_3_‐doped Sb_2_S_3_ were also confirmed using EDX data, which were derived from the HRTEM results (Figures S7 and S8, Supporting Information). The replacement of Sb with Bi could reduce the strains in the lattice of the 2.5 mol% BiI_3_‐doped Sb_2_S_3_ sample. To investigate the effect of this replacement on the interfacial strains, we performed XRD measurements to carry out W‐H analysis. Considering that Sb_2_S_3_ and 2.5 mol% BiI_3_‐doped Sb_2_S_3_ are anisotropic, the stress can be calculated as follows^[^ ^14^ ^]^

(1) $$\begin{array}{c} \beta \cos\theta =\frac{K\lambda }{D}+\left(4\sin\theta {\left(2u/E\right)}^{1/2}\right) \end{array}$$

**Figure 3:**
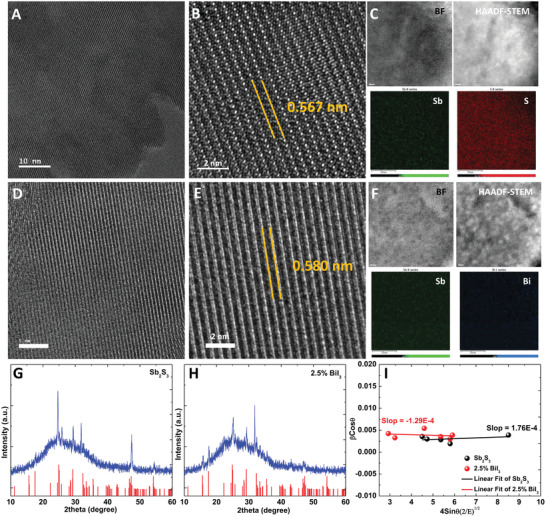
Morphology and strain. A) Low‐magnification and B) high‐magnification high‐resolution transmission electron microscopy (HRTEM), C) bright field high angle annular dark field scanning transmission electron microscopy (BF/HAADF‐STEM) and corresponding elemental mapping images of Sb_2_S_3_. D) Low‐magnification and E) high‐magnification HRTEM, F) BF/HAADF‐STEM and corresponding elemental mapping images of 2.5 mol% BiI_3_‐doped Sb_2_S_3_. XRD patterns of G) Sb_2_S_3_ and H) 2.5 mol% BiI_3_‐doped Sb_2_S_3_ on the glass. The standard Sb_2_S_3_ (JCPDS No. 42–1393) structure file is plotted as the black column. I) Plot of *β* cos *θ* versus 4 sin *θ* (2/*E*)^1/2^ of Sb_2_S_3_ and 2.5 mol% BiI_3_‐doped Sb_2_S_3_.

where *β* is the broadening of peaks, 2*θ* is the actual scattering angle, *K* is shape factor (0.9), *D* is crystalline size, *λ* is wavelength of Cuk_*α*_ radiation, *u* is the energy density (energy per unit volume), and *E* is the Young's modulus (TPa). According to Hooke's law, *u* can be calculated using the equation^[^ ^14^ ^]^

(2) $$\begin{array}{c} u=\varepsilon^{2}E/2 \end{array}$$

Therefore, the slope of the line plotted between *β* cos *θ* and 4 sin *θ* (2/*E*)^1/2^ can be used to calculated u, and the lattice strain can be obtained if the E of the sample is known. Because the content of Bi in 2.5 mol% BiI_3_‐doped Sb_2_S_3_ was very low, the *E* value of Sb_2_S_3_ (71.62 GPa) were used in this study.^[^ ^15^ ^]^ In the light of the overlap of the main XRD peak (24.9°) in the Sb_2_S_3_ and the main XRD peak (25.3°) in TiO_2_, we prepared the glass/Sb_2_S_3_ (2.5 mol% BiI_3_‐doped Sb_2_S_3_) samples to analyze the strain. The *ε* values of Sb_2_S_3_ and 2.5 mol% BiI_3_‐doped Sb_2_S_3_ were calculated to be 1.76 × 10^−4^ and −1.29 × 10^−4^, respectively (Figure 3G–I). The positive and negative values indicated tension and compression strain, respectively. These results indicated that Sb_2_S_3_ bore tension strain, while 2.5 mol% BiI_3_‐doped Sb_2_S_3_ bore compression strain. After incorporating 2.5 mol% BiI_3_ into Sb_2_S_3_, the strain absolute value decreased. This change trend can be reproduced (Figure S9, Supporting Information). Besides, compared to tension strain, a mild compression strain can reduce the bandgap, enhance the carrier lifetime, suppress the vacancy formation, and improve the stability.^[^ ^4^, ^16^ ^]^ In addition, the incorporation of 2.5 mol% BiI_3_ into Sb_2_S_3_ forms the alloy, which reduces the melting point.^[^ ^17^ ^]^ Thus, the compound can form at a lower temperature and in a shorter annealing process, which might reduce the lattice strain. The higher residual stress indicated that the crystals bore more significant lattice distortion, which resulted in more defects and traps in the lattice. Therefore, reducing the residual stress should be beneficial for defect reduction. Thus, the improvement in the performance of solar cells via the incorporation of BiI_3_ into Sb_2_S_3_ could be attributed to the defect reduction induced by the decrease in residual stress.

To further confirm the effect of the decrease in strain on the trap state, the defect energy levels of Sb_2_S_3_ and 2.5 mol% BiI_3_‐doped Sb_2_S_3_ were analyzed by measuring their temperature‐dependent dark current–voltage curves in the temperature range of 25–150 °C with a step of 25 °C (**Figure** **4**A,B). The average activation energy of the trapped electrons can be calculated using the Richardson–Dushman equation^[^ ^18^ ^]^

(3) $$\begin{array}{c} J\propto e^{-\frac{\Delta E}{kT}} \end{array}$$

where *ΔE*, *k*, and *T* are the electron activation energy, Boltzmann constant, and absolute temperature, respectively. Figure 4C depicts the temperature dependence of the dark currents of the Sb_2_S_3_ and 2.5 mol% BiI_3_‐doped Sb_2_S_3_ solar cells. The slopes of the fitted lines can be used to calculate the activation energies. Because the dark current under short‐circuit conditions is significantly affected by noise, we used the dark current at a bias of −0.5 V to calculate *ΔE*. The *ΔE* values of the Sb_2_S_3_ and 2.5 mol% BiI_3_‐doped Sb_2_S_3_ solar cells in the dark were 0.345 and 0.256 eV, respectively. Therefore, 2.5 mol% BiI_3_ doping lowered the trap state energy level, which effectively passivated the trap states. To describe the effect of deep‐level traps in the Sb_2_S_3_ solar cells and low‐level traps in the 2.5 mol% BiI_3_‐doped Sb_2_S_3_ solar cells on the charge transfer and recombination, we drew the schematic diagrams (Figure 4D,E). The photon‐generated carriers produced by light can transfer to the hole transporting layer and electron transporting layer, respectively. According to our previous work, the traps in Sb_2_S_3_ are mainly hole‐like traps.^[^ ^8f^ ^]^ Due to the existence of deep level traps close to the VBMs, holes will be trapped by the deep level traps, and charge recombination might occur between these holes and electrons in the CBMs, which is detrimental to the device performance. In the case of the 2.5 mol% BiI_3_‐doped Sb_2_S_3_ solar cells, holes located in the low‐level traps can still transfer to the hole transporting layer, which will not affect the device performance.

**Figure 4:**
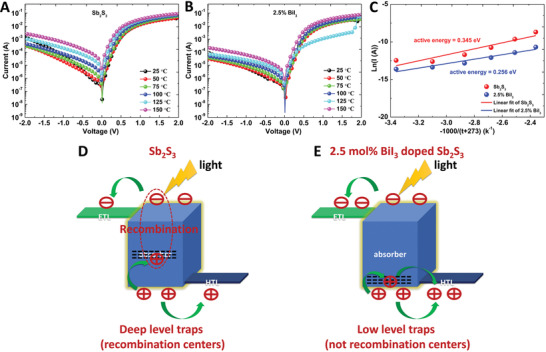
Defect energy level. Temperature‐dependent dark current–voltage curves of A) Sb_2_S_3_ and B) 2.5 mol% BiI_3_‐doped Sb_2_S_3_ solar cells. C) Temperature dependence of the dark currents at the bias of −0.5 V for Sb_2_S_3_ and 2.5 mol% BiI_3_‐doped Sb_2_S_3_ solar cells. D,E) Schematic diagrams to illustrate charge transfer and recombination in the Sb_2_S_3_ and 2.5 mol% BiI_3_‐doped Sb_2_S_3_ solar cells.

The codoping effect obtained from Sb_2_S_3_ has been extended to Sb_2_(S*_x_*Se_1−_ *_x_*)_3_. We used 1,1‐dimethyl‐2‐selenourea as the Se source to add 20 mol% Se to Sb_2_S_3_ and obtained Sb_2_(S*_x_*Se_1−_ *_x_*)_3_ (Figure S10, Supporting Information). The *J*–*V* curves under AM 1.5 G illumination conditions (100 mW cm^−2^) of the best‐performing Sb_2_(S*_x_*Se_1–_ *_x_*)_3_ and 2.5 mol% BiI_3_‐doped Sb_2_(S*_x_*Se_1−_ *_x_*)_3_ solar cells are illustrated in **Figure** **5**A. After doping, the PCE increased from 6.76% to 7.05%, and the *J*_SC_, *V*_OC_, and FF of the 2.5 mol% BiI_3_‐doped Sb_2_(S*_x_*Se_1−_ *_x_*)_3_ solar cell were 21.5 mA cm^−2^, 520.5 mV, and 63.0%, respectively. Figure 5B presents the IPCE spectrum of the 2.5 mol% BiI_3_‐doped Sb_2_(S*_x_*Se_1−_ *_x_*)_3_ solar cell. Integrated *J*_SC_ from the IPCE spectrum produces a current density of 20.30 mA cm^−2^, which was in good agreement with the photocurrent density obtained from the *J*–*V* curve. Figure 5C presents the stabilized current density and PCE of the 2.5 mol% BiI_3_‐doped Sb_2_(S*_x_*Se_1−_ *_x_*)_3_ solar cell. At a bias of 0.416 V, the champion device presented a stabilized current density of 16.48 mA cm^−2^ and stabilized PCE of 6.85%, which indicated the reliability of the obtained PCE values. Figure 5D depicts the histogram of the PCEs of the 43 solar cells analyzed in this study, which was a normal distribution curve.

**Figure 5:**
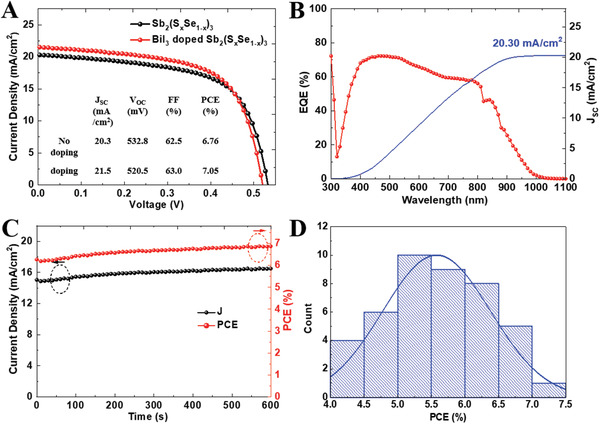
Sb_2_(S*_x_*Se_1−_ *_x_*)_3_‐based solar cells. A) *J*–*V* curves under standard illumination conditions (100 mW cm^−2^) of AM 1.5 G of the best‐performing Sb_2_(S*_x_*Se_1−_ *_x_*)_3_ and 2.5 mol% BiI_3_‐doped Sb_2_(S*_x_*Se_1−_ *_x_*)_3_ solar cells and B) incident photon‐to‐current‐efficiency spectrum of the best‐performing 2.5 mol% BiI_3_‐doped Sb_2_(S*_x_*Se_1−_ *_x_*)_3_ solar cell. C) Maximum power point tracking (0.416 V) of 2.5 mol% BiI_3_‐doped Sb_2_(S*_x_*Se_1−_ *_x_*)_3_ solar cell, measured with the nonencapsulated best‐performing solar cell under full solar illumination (AM 1.5 G, 100 mW cm^−2^ under ambient conditions). D) PCE histogram for the 43 independently 2.5 mol% BiI_3_‐doped Sb_2_(S*_x_*Se_1−_ *_x_*)_3_ solar cells fabricated in this study. Here *J*_SC_, *V*_OC_, FF, PCE, and EQE denote the short‐circuit current density, open‐circuit voltage, fill factor, power conversion efficiency, and external quantum efficiency, respectively.

The humidity, thermal, and photo stability of the Sb_2_S_3_ and 2.5 mol% BiI_3_‐doped Sb_2_S_3_ solar cells were compared. To test the humidity stability, the nonencapsulated cells were stored in a sealed opaque box under ambient conditions: 60% relative humidity and room temperature. As illustrated in **Figure** **6**A, the Sb_2_S_3_ and 2.5 mol% BiI_3_‐doped Sb_2_S_3_ solar cells retained 43.9% and 74.0% of their initial PCEs, respectively, after 360 h of storage. Figure 6B presents the corresponding thermal stability of the solar cells. The Sb_2_S_3_ and 2.5 mol% BiI_3_‐doped Sb_2_S_3_ cells retained 56.2% and 95.6% of their initial PCEs, respectively, after 360 h of exposure to 85 °C air with 20% average relative humidity. In addition, to determine their photo stability, the nonencapsulated solar cells were illuminated under standard AM 1.5 G conditions (100 mW cm^−2^) in the absence of a UV filter at room temperature. As presented in Figure 6C, the Sb_2_S_3_ and 2.5 mol% BiI_3_‐doped Sb_2_S_3_ solar cells retained 21.7% and 34.5% of their initial PCEs, respectively, after illumination for 1450 min. These results indicated that the humidity, thermal, and photo stability of the analyzed solar cells significantly improved after the incorporation of 2.5 mol% BiI_3_ into Sb_2_S_3_, and that might have been due to the decrease in the defects owing to the reduced strain.

**Figure 6:**
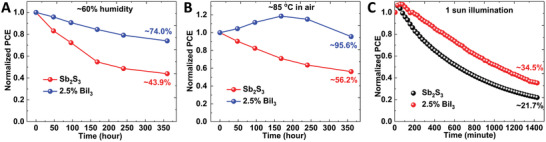
Stability. Time evolution of normalized power conversion efficiency (PCE) of the nonencapsulated Sb_2_S_3_ and 2.5 mol% BiI_3_‐doped Sb_2_S_3_ solar cells: A) under ambient atmosphere (≈60% humidity), in the dark, at room temperature, B) at 85°C in air, under 20% average relative humidity, C) under the standard AM 1.5G illumination of a Xe lamp without an ultraviolet filter.

In summary, strain tuning in Sb‐Chs by simultaneously replacing Sb and S with larger Bi and I ions was demonstrated. Bi/I codoped Sb_2_S_3_ with various Bi/I amounts were prepared using the solution process and precursors that consisted of a Sb‐TU complex and BiI_3_. The bandgap of Bi/I codoped Sb_2_S_3_ decreased within the experimental range of 0–5 mol% of BiI_3_. The tension strain in Sb_2_S_3_ is 1.76 × 10^−4^, and it changes into a relatively smaller compression strain (−1.29 × 10^−4^) in the 2.5 mol% BiI_3_‐doped Sb_2_S_3_. The 2.5 mol% BiI_3_‐doped Sb_2_S_3_ lowered the trap state energy level and effectively passivated the trap states. The PCE of the 2.5 mol% BiI_3_‐doped Sb_2_(S*_x_*Se_1−_ *_x_*)_3_ solar cell was 7.05%. The 2.5 mol% BiI_3_‐doped Sb_2_S_3_ solar cells also presented higher humidity, thermal, and photo stability than the Sb_2_S_3_ solar cells. This study provides a method that could simultaneously tune the bandgaps and relax the residual stress of high‐efficiency metal chalcogenide solar cells.

## Experimental Section

##### Preparation of FTO/TiO_2_‐BL/mp‐TiO_2_

The FTO glass was ultrasonically cleaned with acetone, detergent water, and ethanol, in sequence, for 10 min each. Next, the FTO glass was subjected to spray pyrolysis using 20 × 10^−3^ m titanium diisopropoxide bis(acetylacetonate) (Aldrich) solution at 450 °C to deposit a 100 nm thick TiO_2_ blocking layer (TiO_2_‐BL) on it. A TiO_2_ paste consisting of anatase TiO_2_ nanoparticles (average diameter of 50 nm) was screen‐printed onto the FTO/TiO_2_‐BL substrate to form 150 to 1800 nm thick mp‐TiO_2_ layers. Subsequently, the samples were calcined at 500 °C for 1 h in air to crystallize TiO_2_.

##### Preparation of FTO/TiO_2_‐BL/mp‐TiO_2_/Bi/I codoped Sb_2_S_3_

The precursor of Bi/I codoped Sb_2_S_3_ was prepared by dissolving SbCl_3_ (0.5–1.5 mmol) and thiourea (thiourea/SbCl_3_ mole ratio of 0.5–1.8:1) in 1 mL *N*,*N*‐dimethylformamide (DMF); then, 0–5 mol% BiI_3_ was added to the solution. The precursors were spin‐coated on the FTO/TiO_2_‐BL/mp‐TiO_2_ substrates at 1000–2000 rpm for 1 min. Subsequently, the substrates were thermally decomposed in Ar at 200 °C for 5 min and annealed in Ar at 300 °C for 5 min. Finally, the samples were washed in DMF to remove the residual BiI_3_.

##### Device Fabrication

A solution of 10 mg PCPDTBT and 10 mg PC_71_BM (Nano C 1, 2‐dichlorbenzene) in 1 mL 1, 2‐dichlorobenzene was spin‐coated onto FTO/TiO_2_‐BL/mp‐TiO_2_/Bi/I codoped Sb_2_S_3_ at 2500 rpm for 60 s to obtain the hole transporting material. Subsequently, poly(3,4‐ethylenedioxythiophene) codoped with poly(4‐styrenesulfonate) (PEDOT:PSS; Baytron AI 4083), which was diluted threefold in methanol, was spin‐coated at 2500 rpm for 60 s to form the hole transporting layer. Lastly, Au was deposited onto the samples, as the anode. The active area of each device was 16 mm^2^.

##### Material and Device Characterization

The XRD profiles and ultraviolet–visible (UV–vis) absorption spectra of the samples were acquired using a D/MAX2500V/PC (Rigaku, Japan) powder X‐ray diffractometer, and a V‐780 (Jasco, USA), UV–vis spectrophotometer, respectively. An SU‐8220 (Hitachi, Japan) FE‐SEM device was used to analyze the morphology and record the EDX profiles of the samples. An ESCLAB 250XI (Thermo‐Fischer, USA) with a monochromatic Al K*α* source (1486.6 eV) or an unfiltered He I (21.22 eV) gas discharge lamp was used to acquire the XPS and UPS profiles of the samples, respectively. An AUT302N (Metrohm Autolab, USA) instrument was used for EIS measurements. A JEM‐2100F (JEOL, Japan) HRTEM system was used to investigate the microscopic structure of the specimens. A Titan3 G2 60–300 (FEI, USA) instrument equipped with a probe‐side spherical aberration (Cs) corrector was used to obtain the HAADF‐STEM images and perform EDX measurements. A commercial FluoTime 300 (PicoQuant GmbH, Germany) time‐correlated single photon counting setup (equipped with a PMA‐C‐192‐M detector and high‐resolution excitation monochromators was used to obtain the steady‐state PL and TRPL spectra of the samples. A 520 nm laser was used to excite the samples. A 550 nm cut‐off filter was used to filter the undesired light. The PL spectra from 600 nm to 900 nm were detected. TRPL spectra were collected at the wavelength of the maximum PL intensity. The samples were coated on the glass/Al_2_O_3_ substrates. The TRPL spectra were fitted using the following equation: $y=A_{1}\exp\left(\frac{t}{t_{1}}\right)+y_{0}$, where y is the time‐dependent emission intensity, A_1_ is the relative amplitudes, and t_1_ is the fitted PL lifetime. The *J*–*V* curves were obtained using an Oriel Class A, 91195A (Newport, USA) solar simulator with a Keithley 2400 source meter at 100 mA cm^−2^ illumination AM 1.5 G. If not mentioned, the *J*–*V* curves were measured in reverse scan mode. The step voltage was 10 mV, and the delay time was 40 ms. The forward scan mode was from −0.5 to 1.0 V, and the reverse scan mode was from 1.0 to −0.5 V. The delay time was set at each voltage step before the current was measured. The *J*–*V* curves of solar cells were measured by using a light mask to make the active area be 0.096 cm^2^. The IPCE spectra were measured using an Oriel, IQE 200B (Newport, USA) internal quantum efficiency system.

##### Statistical Analysis

For the stability test, the PCE was normalized. The strain value and activation energy were obtained by the linear fitting. The impedance spectra were fitted using a Z‐View software. The carrier lifetime data were fitted by an exponential function. The sample sizes for XRD, XPS, UPS, and UV–vis absorption measurements were 1.5 × 1.5, 1.5 × 1.5, 1.5 × 1.5, and 2.5 × 2.5 cm. The solar cell size was 2.5 × 2.5 cm. Most data were processed using the origin software.

## Conflict of Interest

The authors declare no conflict of interest.

## Supporting information

Supporting Information
