# Supplementary material for: Strain Tuning via Larger Cation and Anion Codoping for Efficient and Stable Antimony‐Based Solar Cells
Source: Adv Sci (Weinh). 2020 Nov 23;8(1):2002391. doi: 10.1002/advs.202002391 (PMC7788500; doi:10.1002/advs.202002391)
Supplement: Supplementary file 1 — Supporting Information [file ADVS-8-2002391-s001.pdf]

© 2020 Wiley-VCH GmbH

ADVANCED SCIENCE
Open Access

Supporting Information

for *Adv. Sci.*, DOI: 10.1002/advs.202002391

Strain Tuning via Larger Cation and Anion Co-Doping for Efficient and Stable Antimony-Based Solar Cells

*Riming Nie, Kyoung Su Lee, Manman Hu, and Sang Il Seok**

Supporting Information

Strain Tuning via Larger Cation and Anion Co-Doping for Efficient and Stable Antimony-Based Solar Cells

*Riming Nie, Kyoung Su Lee, Manman Hu, and Sang Il Seok**

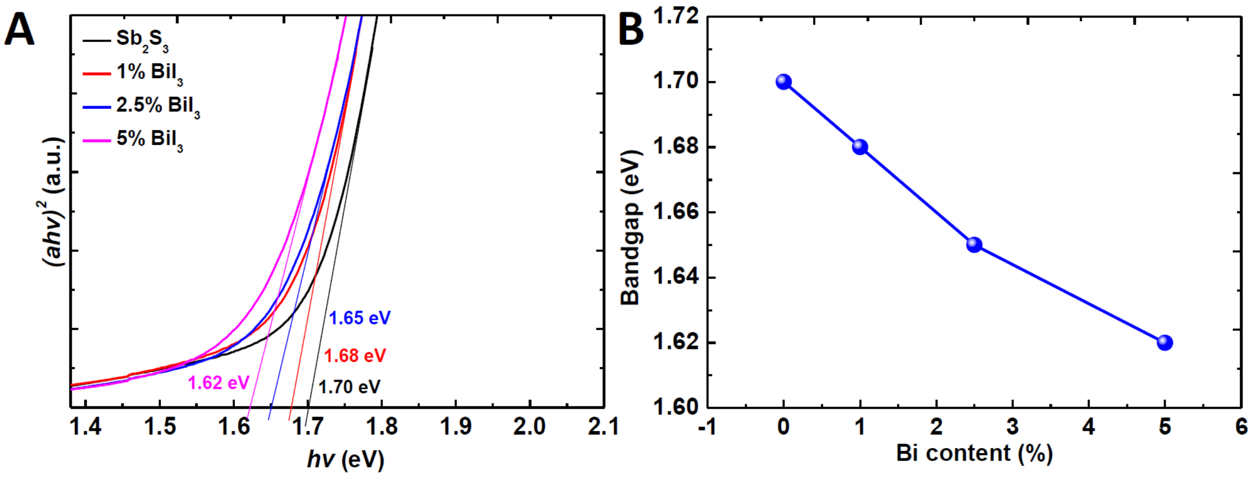

Figure S1. Optical properties. (**A**) Tauc plots of the glass/mesoporous TiO_2/Sb_2S_3 (Bi/I co-doped Sb_2S_3) cells with Bi content in the range of 0–5 mol%. (**B**) Relationship between the bandgaps of Bi/I co-doped Sb_2S_3 and Bi content.

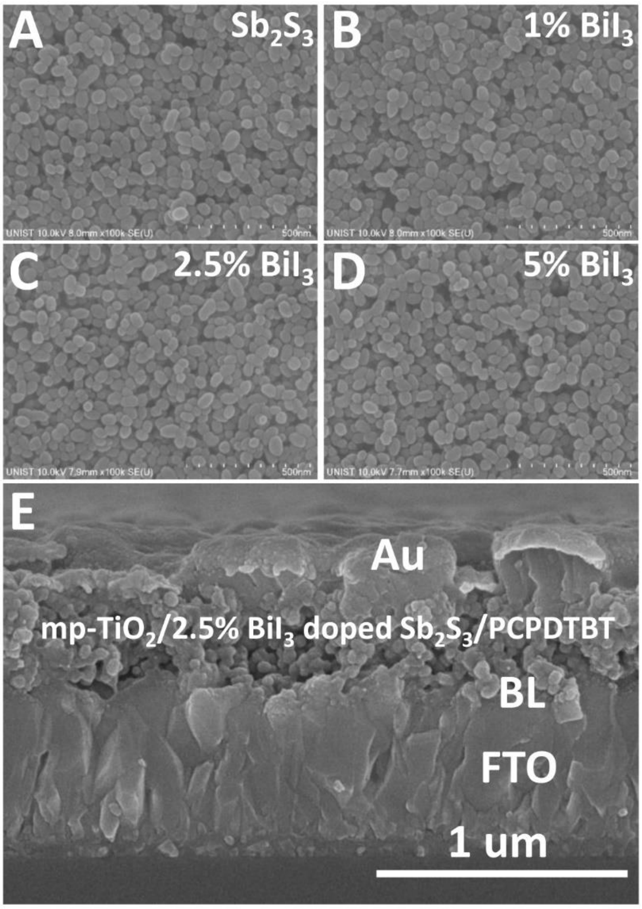

Figure S2. Surface Morphology and Distribution. Surface field-emission scanning electron microscopy (FE-SEM) images of the glass/ mesoporous TiO_2 (mp-TiO_2)/Sb_2S_3 (Bi/I co-doped Sb_2S_3) cells: (**A**) Sb_2S_3, (**B**) 1 mol% BiI_3-, (**C**) 2.5 mol% BiI_3-, and (**D**) 5 mol% BiI_3-doped Sb_2S_3. (**E**) Cross-sectional FE-SEM image of the FTO/blocking layer (BL)/mp-TiO_2/2.5 mol% BiI_3-doped Sb_2S_3)/hole-transporting material and layer/Au cells. Here PCPDTBT denotes poly [2,6-(4,4-bis(2-ethylhexyl)-4H-cyclopenta[2,1-b;3,4-b′]dithiophene)-alt-4,7-(2,1,3-benzothiadiazole)].

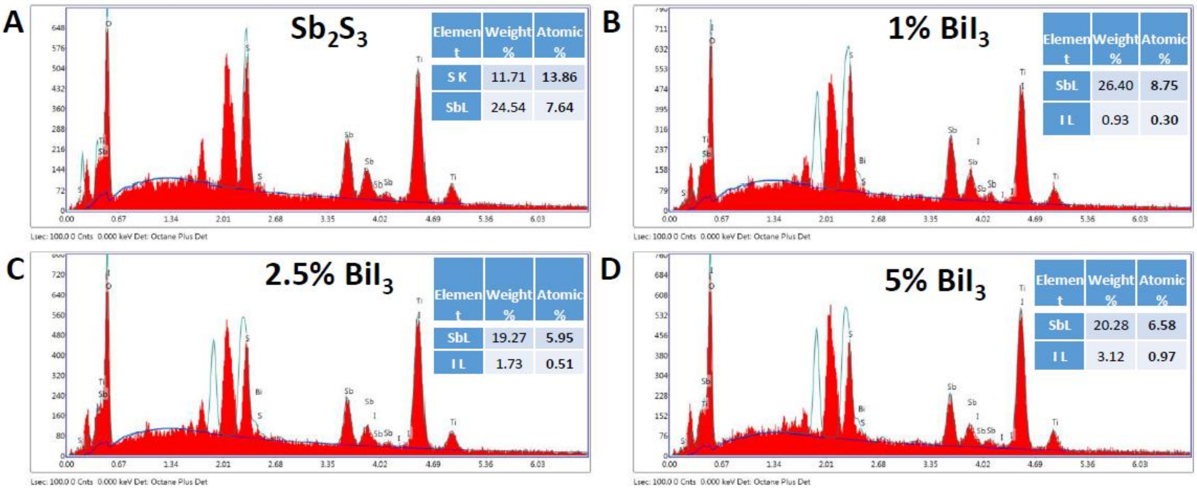

Figure S3. Energy-dispersive X-ray spectroscopy data of the glass/mesoporous TiO_2 /Sb_2S_3 (Bi/I co-doped Sb_2S_3). (**A**) Sb_2S_3, (**B**) 1 mol% BiI_3-, (**C**) 2.5 mol% BiI_3- and (**D**) 5 mol% BiI_3-doped Sb_2S_3.

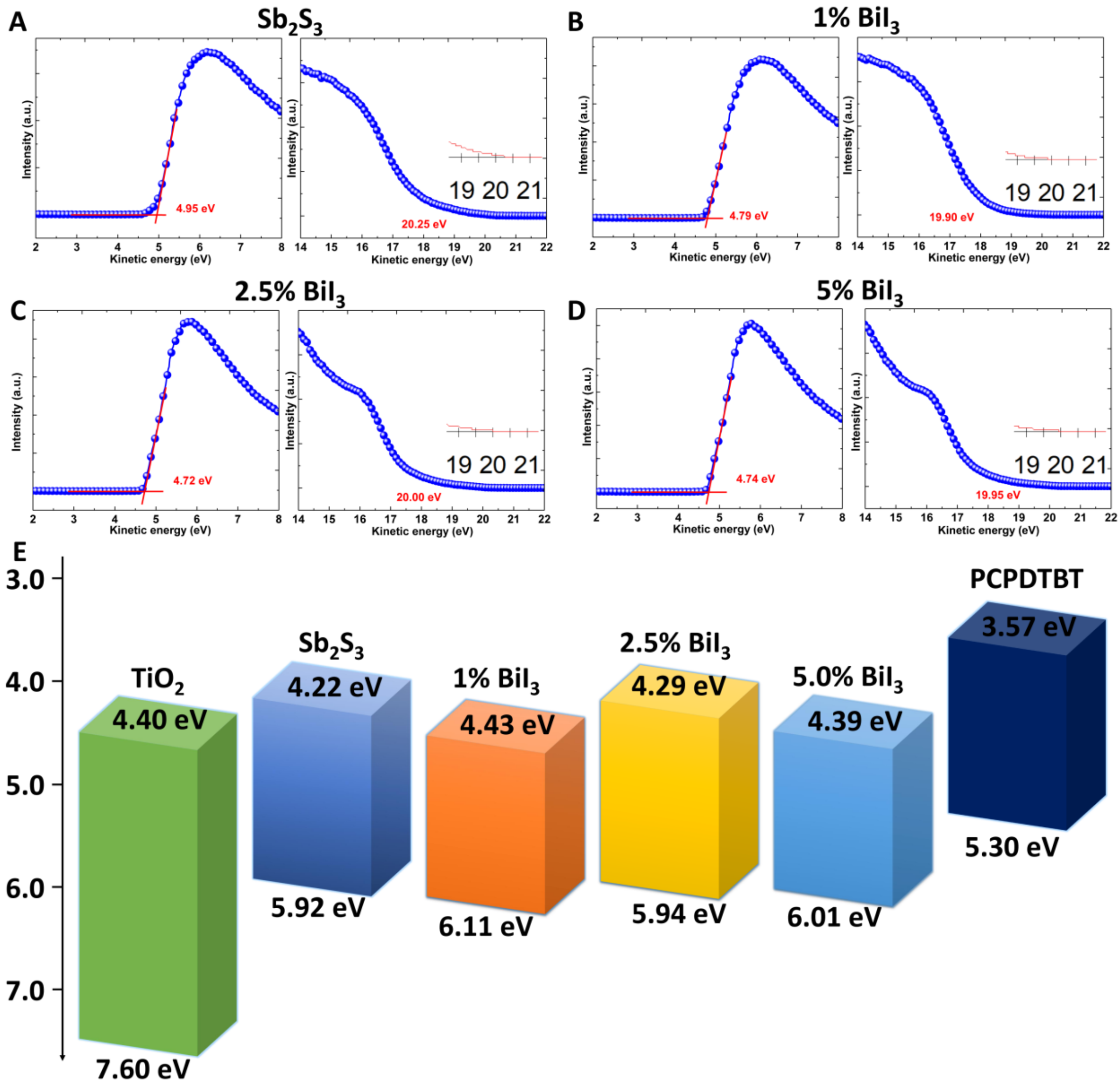

Figure S4. Energy levels. The secondary electron cutoff and the highest molecular orbital (HOMO) regions of HeI UPS spectra for the FTO/mp-TiO_2/Sb_2S_3 (Bi and I co-doped Sb_2S_3). (**A**) Sb_2S_3, (**B**) 1% BiI_3, (**C**) 2.5% BiI_3, and (**D**) 5% BiI_3 doped Sb_2S_3. Insets are the magnified images of the highest molecular orbital (HOMO) regions. (**E**) The energy level illumination of Sb_2S_3 (Bi and I co-doped Sb_2S_3), TiO_2 and PCPDTBT.

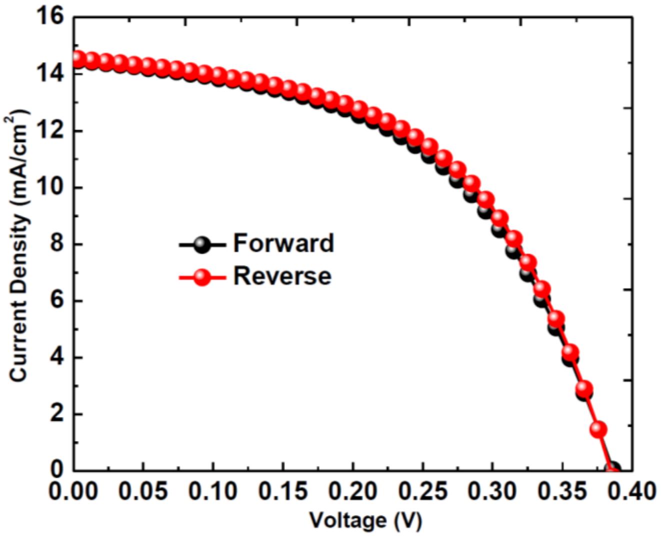

Figure S5. **J-V Hysteresis behavior of the device.** J-V curves of the 2.5 mol% BiI_3 doped Sb_2S_3 solar cell measured under AM 1.5 (100 mW/cm^2) with forward and reverse scan modes. The forward scan mode is from -0.5 V to 1.0 V, and the reverse scan mode is from 1.0 V to -0.5 V. The voltage step and delay time are 10 mV and delay time of 40 ms).

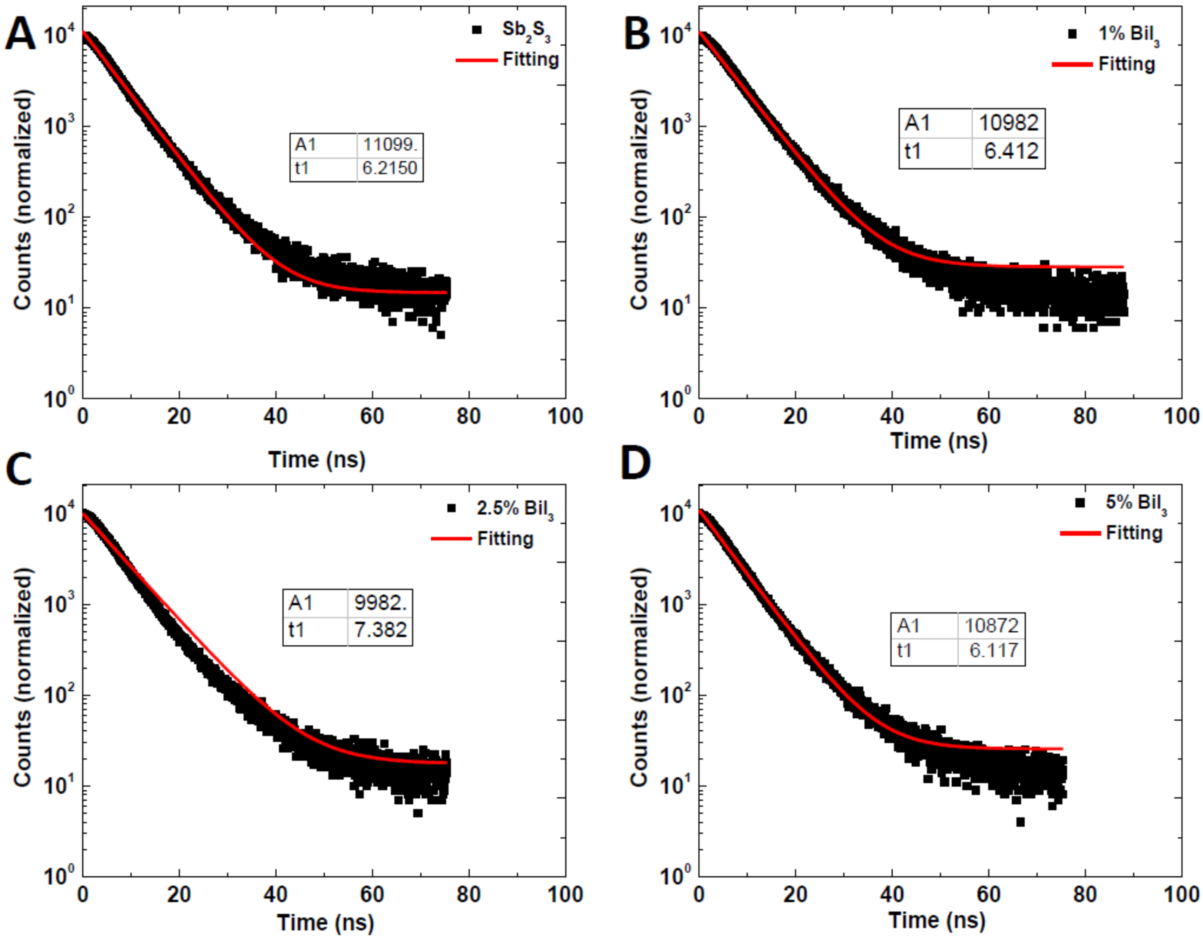

Figure S6. **Time-resolved photoluminescence (TRPL) profiles of Sb_2S_3 and Bi/I co-doped Sb_2S_3**. (**A**) Sb_2S_3, (**B**) 1 mol% BiI_3-, (**C**) 2.5 mol% BiI_3-, and (**D**) 5 mol% BiI_3-doped Sb_2S_3. The TRPL data is fitted using an equation of $y = A_1*\exp(-t/t_1) + y_0$. Here t_1 is the PL lifetime.

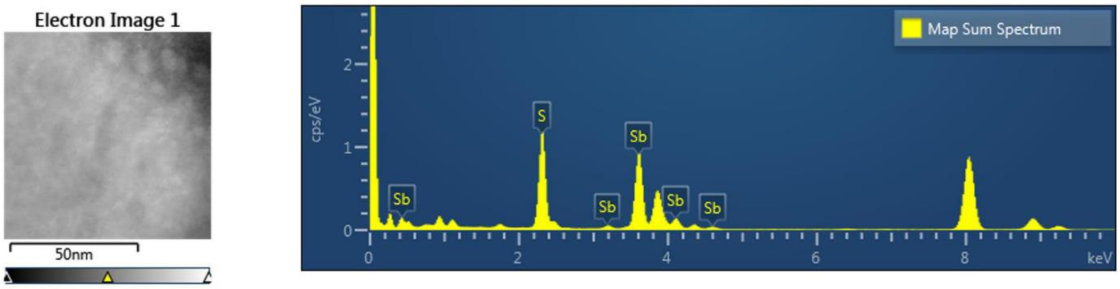

Element	Line Type	k Factor	k Factor type	Absorption Correction	Wt%	Wt% Sigma	Atomic %
S	K series	0.994		1.00	24.68	0.17	55.44
Sb	L series	1.940		1.00	75.32	0.17	44.56
Total:					100.00		100.00

Figure S7. Energy-dispersive X-ray spectroscopy data of Sb_2S_3 derived from the high-resolution transmission electron microscopy results.

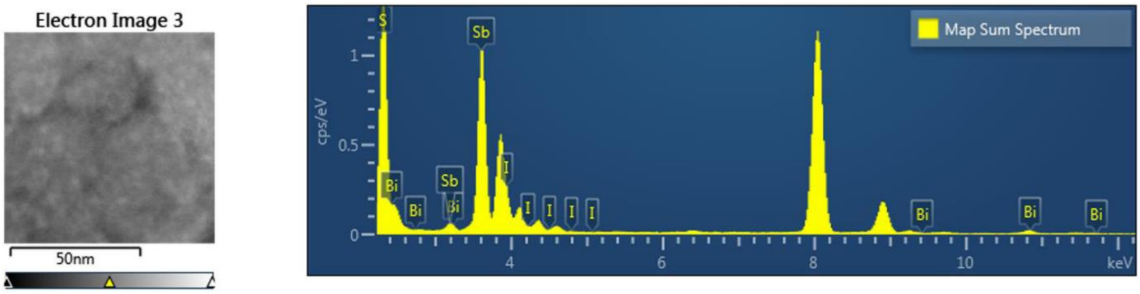

Element	Line Type	k Factor	k Factor type	Absorption Correction	Wt%	Wt% Sigma	Atomic %
S	K series	0.994		1.00	25.16	0.28	56.36
Sb	L series	1.940		1.00	71.41	0.50	42.13
I	L series	1.969		1.00	1.49	0.37	0.84
Bi	M series	1.805		1.00	1.94	0.48	0.67
Total:					100.00		100.00

Figure S8. Energy-dispersive X-ray spectroscopy data of 2.5 mol% BiI_3-doped Sb_2S_3 derived from the high-resolution transmission electron microscopy results.

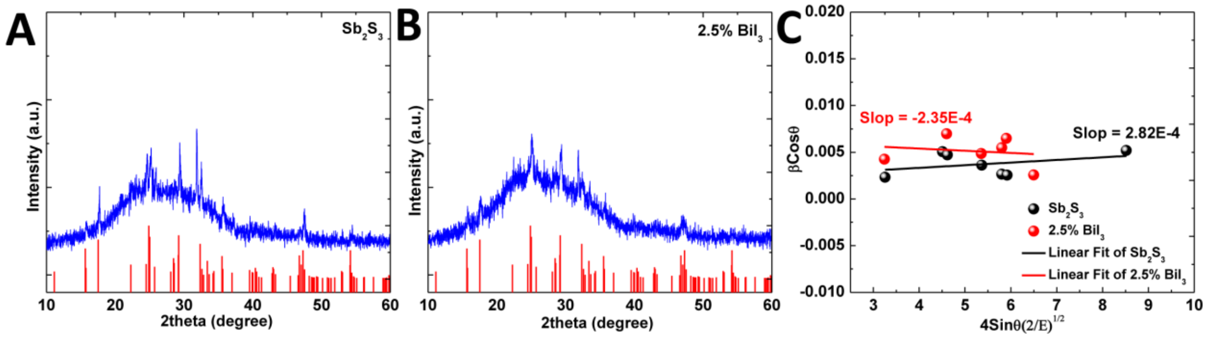

Figure S9. XRD patterns of (A) Sb_2S_3 and (B) 2.5 mol% BiI_3-doped Sb_2S_3 on the glass. The standard Sb_2S_3 (JCPDS No. 42-1393) structure file is plotted as the black column. (C) Plot of $\beta\cos\theta$ vs $4\sin\theta(2/E)^{1/2}$ of Sb_2S_3 and 2.5 mol% BiI_3-doped Sb_2S_3.

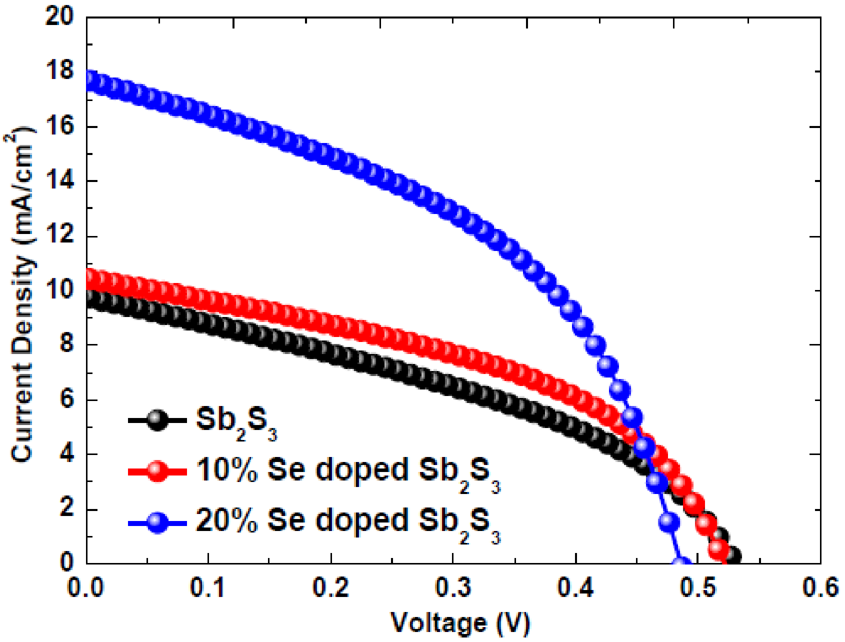

Figure S10. Performance of $Sb_2(S,Se)_3$ solar cells. J–V curves under standard illumination conditions (100 mW cm^{-2}) of AM 1.5 G of the Sb_2S_3 solar cells with and without added Se. The Se source was 1,1-dimethyl-2-selenourea.

Table S1. Performance of the Sb_2S_3- and Bi/I co-doped Sb_2S_3-based solar cells. Here J_{SC}, V_{OC}, FF, and PCE denote the short-circuit current density, open-circuit voltage, fill factor, and power conversion efficiency, respectively.

	J_{SC} (mA/cm^2)	V_{OC} (mV)	FF (%)	PCE (%)
Sb_2S_3	12.08	487	39.8	2.34
1 mol% BiI_3	13.75	482	49.8	3.30
2.5 mol% BiI_3	14.87	468	53.0	3.69
5 mol% BiI_3	6.63	393	38.8	1.01

Table S2. Data extracted from Figure 2D. Here, R_s denotes the series resistance. R_{trans}/C_{trans} denote the resistance/capacitance associated with HTM. R_{rec}/C_{rec} denote the resistance/capacitance related to the Bi/I co-doped Sb_2S_3/HTM interface.

	R_s (Ω)	R_{trans} (Ω)	C_{trans} (F)	R_{rec}	C_{rec} (F)
Sb_2S_3	42.16	157.2	1.06×10^{-7}	7099	3.11×10^{-7}
1 mol% BiI_3	21.28	117.8	4.01×10^{-7}	4494	2.59×10^{-7}
2.5 mol% BiI_3	39.26	62.03	1.66×10^{-7}	3905	2.79×10^{-7}
5 mol% BiI_3	20.16	235.3	3.79×10^{-7}	1798	4.77×10^{-7}
